# Phosphorus and nitrogen-containing soybean oil polyols: Effect on the mechanical properties and flame retardancy of polyurethane foam

**DOI:** 10.55730/1300-0527.3656

**Published:** 2024-01-22

**Authors:** Deniz ÖZTAŞKIN, Lütfullah Yusuf YİVLİK, İlayda ACAROĞLU DEGITZ, Tarık EREN

**Affiliations:** 1 Department of Chemistry, Faculty of Arts and Science, Yıldız Technical University, İstanbul, Turkiye; 2 Flokser Chemical Company, İstanbul, Turkiye

**Keywords:** Epoxidized soybean oil, renewable resources, phosphorus, flame retardancy, polyurethane foam

## Abstract

In recent years, there has been an increasing interest in producing new materials that use renewable resources and halogen-free flame retardants with nonleaching properties. This research focuses on designing and synthesizing phosphorus-nitrogen-based biopolyols for use in polyurethane (PU) foam production. Polyol (ESBO-DYM) with dual functionalities, renewability, and nonflammability is synthesized through the epoxy ring-opening reaction of epoxidized soybean oil with phosphorus and nitrogen-containing tetraol products (DYM). The mechanical and flame retardant properties of PU foams with the addition of an ESBO-DYM were investigated. Increasing the amount of phosphorus in the PU foams increased the thermal stability properties. Using 100% ESBO-DYM as a polyol in the foam formulation increased the limiting oxygen index (LOI) value to 22.9% and resulted in the highest char yield according to the thermal gravimetric analysis (17% at 600 °C). Additionally, the introduction of ESBO-DYM polyol into the formulation resulted in a decrease in the compression strength of the foams. The foam density decreased as the amount of ESBO-DYM polyol in the formulation increased. The foam with the highest amount of ESBO-DYM had a foam density of 29.1 kg/m^3^. The morphology of the foams was characterized using a scanning electron microscope (SEM). As a result of this study, flame retardant polyurethane foams were formulated using a renewable source, polyol, along with commercial compounds.

## 1. Introduction

In recent years, the synthesis of polymers from renewable and environmentally friendly sources such as plant and animal oils has gained greater importance due to the drive to minimize the dependency on petroleum derivatives. Plant oil triglycerides as renewable raw materials in polymer synthesis provide many advantages due to their biodegradability, CO_2_ emission reduction [1], energy savings, ease of production [2], relatively low cost [3], and the presence of various chemical transformations on triglycerides [4,5] as compared to the petroleum-based products. Among the natural oils, soybean oil is of particularly great interest due to its high availability and low cost throughout the world. Soybean oil is a highly used raw material in the chemical industry due to having a high level of unsaturation (iodine number 140) which allows it to be highly reactive and employs different chemical transformations to functionalize the structure [6]. In the last decade, modified vegetable oils, especially soybean oil, have been extensively used in the production of polymers and composite materials, as well as in many other applications. The reactive regions of soybean oil allow it to be polymerized directly or after modifications of reactive functional groups [7–20]. The synthetic pathway typically involves derivatizing vegetable oils to have polyol functionality; thus, it can be used further in polyurethane and polyester synthesis [21]. However, these polymer matrixes are combustible and do not exhibit flame retardant properties. Therefore, they require an additive or functionalization to resist fire. There are many types of flame retardant materials available in the markets, with the primary target being to maximize the product’s life and properties during fire [22]. To achieve this target, noncombustible and/or fire-retardant materials can be used in the synthesis of polymers. While inorganic additives and halogenated flame retardants play an important role in flame retardancy and smoke suppression, current research focuses on polymeric materials with intrinsic flame retardant properties. These materials do not leach from the matrix, have a long lifespan, and are environmentally friendly. Polymers with inherent flame-retardant properties result in better homogeneity compared to inorganic additives that may leach out of the matrix during processing or over time. Currently, phosphorus-containing reactive or oligomeric flame retardants have become increasingly popular [23–26]. Phosphorus can be incorporated into the polymer matrix through surface modifications, the use of phosphorus-based monomers in polymerization, or blending with high molecular weight additives. The presence of phosphorus in the polymer matrix functions in both the condensed and vapor phases [27–29]. At high temperatures, the formation of polyphosphoric acid and the release of water vapor cause the formation of carbonaceous char and protect the matrix from flame. Additionally, the release of phosphorus-containing radicals prevents the formation of new free radicals and captured oxygen which contributes to the extinction of the flame. Recent reviews have discussed the reactive types of organophosphorus reagents which can be attached to the polymer matrix covalently [27,28]. In the literature, the synthesis of phosphorus-containing polyurethanes derived from vegetable oils and their flame retardant properties have been discussed [30–32]. For example, Zhang et al. synthesized phosphorus-based biodegradable polyols which were used in the polyurethane foam formulation [33]. They reported that the sample containing 3% phosphorus had a limiting oxygen index (LOI) value of 24.3% and the highest char yield (15.97%) when heated to 700 °C. Norzali et al. synthesized phosphorus-bearing polyols from palm oil and used them in polyurethane formulation [34]. They investigated the thermomechanical properties of polyurethanes containing different amounts of phosphate ester-bearing polyols and reported that increasing the amount of phosphorus improved the flame retardant properties, but the mechanical properties were optimal in polyurethane containing 5% phosphorus functional polyol loading. Heinen et al. synthesized phosphorylated polyols from vegetable oil and investigated their flame retardancy [35]. The resulting biobased polyols were used in combination with the polymeric diisocyanate to form polyurethane. Different proportions of phosphorus-based polyols were added to the polyurethane formulation along with other polyols such as castor oil, glycerin, and glycol polyesters. The resulting product was compared with the commercial flame retardants used in the polyurethane formulation. The covalent attachment of phosphorus into the polyurethane matrix resulted in a higher LOI value compared to the polyurethane containing the commercially used flame retardant.

In this study, a phosphorus and nitrogen-containing soy-based polyol (ESBO-DYM) was derived using commercially available compounds: diethanolamine, phenyl phosphonic dichloride, and epoxidized soybean oil (Scheme). When the studies are examined, it has been found that phosphorus-based polyols have been synthesized and applied in polyurethane formulations. It is predicted that the inclusion of a phenyl ring in functional soy polyol, in addition to the synergistic effect of phosphorus and nitrogen, may enhance the formation of char residue. This product was further used in polyurethane foam formulation, as shown in Scheme. The optimum weight ratio of ESBO-DYM polyol was systematically analyzed using thermogravimetric analysis (TGA) and LOI. The synergistic effects of phosphorus and nitrogen on the improvement of the flame retardancy of PU foam formulation were discussed. Additionally, the density and morphological properties of the end product were investigated using scanning electron microscopy (SEM). The newly designed product which provided a biobased and spontaneously flame retardant polyol was covalently attached to PU foam. The objective of this work was to synthesize a phosphorous biobased polyol that can replace petrochemical polyols in polyurethane foam formulation. Consequently, there is no need to add a flame retardant to the formulation again.

## 2. Materials and methods

### 2.1. Materials

Diethanolamine, phenyl phosphonic dichloride, p-toluene sulfonic acid, triethylamine, diethyl ether, acetone, dimethyl sulfoxide (DMSO), dimethyl formamide (DMF), and dichloromethane (DCM) were purchased from Sigma-Aldrich (St. Louis, MO, USA). Epoxidized soybean oil (ESBO, 4.2 epoxy eq.) was donated by Flokser Co. (İstanbul, Türkiye). DMF was distilled prior to analysis. p-toluene sulfonic acid was dried at 70 °C for 2 h. Both ESBO and diethanolamine were dried at 100 °C for 2 h before use. The reagents used for preparing polyurethanes (PUs) were PETOL PZ 400-4G, a polyether polyol with a hydroxyl index of 400 mg KOH/g, supplied by Oltchim (Valcea County, Romania); catalyst N, N-dimethyl cyclohexylamine (DMCHA) and BL-11, supplied by Evonik Industries (Essen, Germany); deionized water; surfactant Tegostab B8462, a polysiloxane polyether copolymer (Evonik Industries); and N, N-diphenylmethane diisocyanate (pMDI) (Desmodur 44V 20L), which contains 31.3% of NCO groups, supplied by Bayer (Leverkusen, Germany).

### 2.2. Instrumentation

The products were characterized using Fourier transform infrared spectroscopy, performed with a PerkinElmer Spectrum I spectrophotometer (PerkinElmer, Waltham, MA, USA) in reflection mode equipped with an ATR diamond module (FTIR-ATR). Additionally, ^1^H NMR (500 MHz), ^13^C NMR (75 MHz), and ^31^P {^1^H} NMR spectra were recorded using a Bruker Avance III 500 MHz spectrometer (Bruker, Rheinstetten, Germany). NMR analyses were conducted using an internal standard tetramethylsilane (TMS) and an external standard CDCl_3_ and *d*_6_-DMSO. Thermal analyses were performed using a Thermogravimetric Analyzer (SII6000 Exstar) (Germany) under an N_2_ atmosphere. The temperature range is from 40 ºC to 600 ºC with a heating rate of 10 ºC /min. Morphological analysis was performed using scanning electron microscopy (SEM, Philips XL30; Philips, Eindhovan, The Netherlands). The static compressive strength of polyurethane foams was determined using an Instron 3366 (Instron, Norwood, MA, USA) type mechanical testing machine in accordance with ISO 844. The measurement of limiting oxygen index (LOI) was conducted using the Fire Testing Technology instrument (Fire Testing Technology, East Grinstead, UK), in accordance with the ASTM D2863-17 standard. The dimensions of the test specimen were 100 mm × 10 mm × 10 mm. The density of the foams was determined according to the ASTM D1622 standard. The hydroxyl value of the soy polyols was determined by the esterification method according to the (ASTM 4274-99). All samples were analyzed twice to determine each property.

### 2.3. Synthesis

#### 2.3.1. Synthesis of phosphorus-based tetraol (DYM)

DYM was synthesized according to a slightly revised procedure outlined in the literature [36,37]. **I**n a two-neck flask, triethylamine (1.21 g, 0.012 mol) and diethanolamine (4.21 g, 0.04 mol) were dissolved in 4 mL of dimethyl sulfoxide. Phenylphosphonic dichloride (1.94 g, 0.01 mol) was then dissolved in 4.86 mL of DMSO and added dropwise to the reaction medium over a period of 30 min. The reaction was stirred under a nitrogen atmosphere for 4 h at −10 °C, followed by continued stirring at room temperature for 24 h. Subsequently, the product was initially extracted using diethyl ether (7 × 30 mL) to remove dimethyl sulfoxide as much as possible. This was followed by extraction with acetone (7 × 30 mL) to remove the residual diethanolamine and the triethylamine salt formed in the reaction mixture. The resulting product was dried in a vacuum oven overnight, and a yellow, viscous product was obtained with a yield of 75–80%.


H1 NMR (DMSO-d6 δ ppm): 7.66-7.35 (aromatic protons,m),3.67 (-OCH2,t),2.99 (-NCH2,t)P31 {H1} NMR (DMSO-d6 δ ppm): 9.38 (1P,s),2.24 (1P,s,formation of phenyl phosphonic acid as an impurity).

#### 2.3.2. Synthesis of phenyl phosphonic acid model compound

A model compound of the phosphoric acid was prepared to determine the second phosphorus peak on the ^31^P NMR spectrum which occurred due to the presence of a byproduct. The phenyl phosphonic dichloride (0.00055 mol) and triethylamine (0.001 mol) were dissolved in a mixture of 0.28 mL *d*_6-_DMSO and 1 mL deuterium water in an NMR tube. The reaction was stirred for 4 h at −10 °C and continued to be stirred at room temperature for 48 h under a nitrogen atmosphere. Following this, the reaction was characterized using ^31^P NMR spectroscopy.


P31 {H1} NMR (DMSO-d6 δ ppm): 2.24

#### 2.3.3. Ring-opening reaction of epoxidized soybean oil with tetraol (ESBO-DYM)

Epoxidized soybean oil (ESBO, 4.87 g, 0.050 mol) and DYM (3.32 g, 0.01 mol) were dissolved in 6 mL of DMF, and *p*-toluene sulfonic acid (0.39 mmol) was added as a catalyst. The reaction was stirred under a nitrogen atmosphere at reflux conditions for 4 h. Subsequently, the reaction mixture was allowed to cool down to room temperature before adding 20 mL of dichloromethane. The resultant product was extracted using a 5% NaCl (5 × 25 mL) solution, and the residual water was removed with sodium sulfate as a drying agent. Sodium sulfate was removed via filtration and the residual solvents were removed using a vacuum oven. The polyol (ESBO-DYM) was obtained with a yield of 80%.


H1 NMR (CDCl3 δ ppm): 7.33-8.11 (aromatic protons,m),4.18-4.13 (-COOCH2),3.72 -3.53 (CHOH),0.89 (-CH3).P31 {H1} NMR (CDCl3 δ ppm): 11.12,12.41,14.93,17.53,and 19.82 ppm.

FTIR (cm^−1^): 3373 (−OH stretching), 3087 (aromatic −CH stretching), 2880 (−CH stretching), 2701 (P=O(OH) weak stretching), 1690 (C=O stretching), 1661 (P=O-N stretching) 1578 (C=C stretching), 1445 (−CH_2_ bending), 1424 (−CH_3_ bending), 1248 (P=O stretching), 1224 (−C-O-C− stretching), 1144 (P=O stretching), 1121 (−CH-OH stretching), 1082 (−CH-OH stretching), 892 (−C=C− bending).

#### 2.3.4. ESBO-DYM polyol-based polyurethane foam formulation

The synthesized phosphorus-containing soybean oil-based polyol (ESBO-DYM) was used in the formulation of rigid PU foam, in conjunction with polyether polyol. Table 1 represents the respective compositions of ESBO-DYM polyol mixed with the other ingredients at 21 ºC. The weight ratio between polyol and polymeric methylene diphenyl diisocyanate (PMDI) was kept constant at 1:1.4 (polyol:isocyanate). Both standard polyether polyol and ESBO-DYM have 400 mg KOH/g, with the polyol:isocyanate ratio kept constant, and the index remaining unchanged. The theoretical OH number for all prepared mixtures is 280 mg KOH/g.

## 3. Results and discussion

### 3.1. Synthesis of tetraol functional phosphonate (DYM)

**Figure 1:**
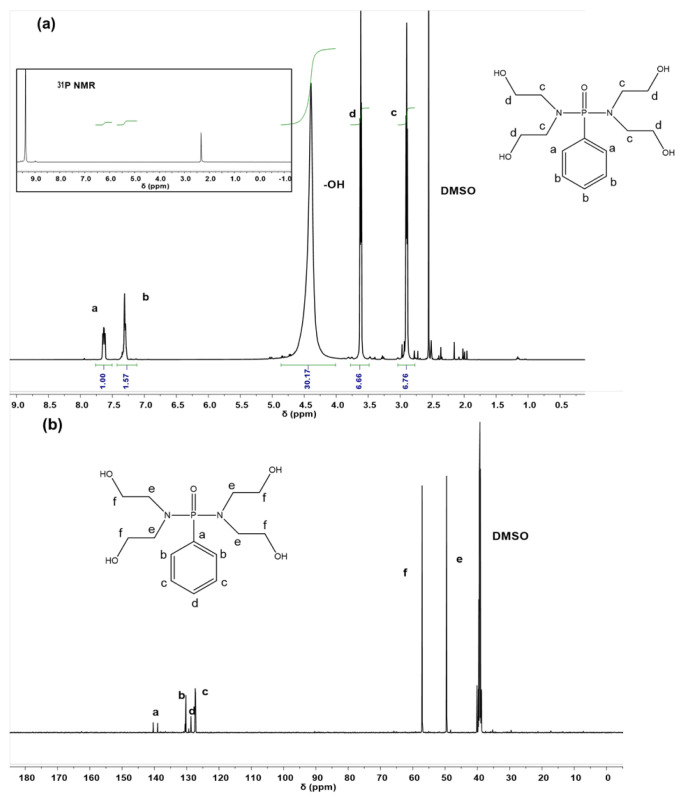
a) ^1^H NMR spectrum of DYM (inset spectrum is ^31^P NMR), b) ^13^C NMR spectrum of DYM.

The synthesis of DYM was followed by the reaction between phenyl phosphonic dichloride with excess diethanolamine at ambient temperature (Scheme). The structures were characterized using ^1^H NMR, ^13^C NMR, and ^31^P {^1^H} NMR techniques. The aromatic ring protons of DYM were observed at 7.66–7.35 ppm on the ^1^H NMR spectrum (Figure 1a). Methylene protons (−OC**H**_2_−) were observed at 3.67 ppm as a triplet with a *J* value of 4.4 Hz. Methylene protons close to the phosphoramide (−NC**H**_2_−) appeared at 2.99 ppm as a triplet with a *J* value of 4.0 Hz.

A deviation was observed in the integration value of the OH proton, which may be attributed to trace amounts of residual water. Theoretically, integration of phenyl protons against to methylene protons (−CH_2_OH) at 3.67 ppm should be 0.63 (5 protons phenyl/8 protons hydroxymethyl); however, the observed integration of phenyl protons against methylene protons (−CH_2_OH) was found to be 0.39 (i.e. 2.57/6.66). The resolution, acquisition, processing parameters, and temperature of the analysis are critical factors for achieving greater accuracy in precise quantification via NMR [38]. The interactions between molecules themselves and the solvent (in this case, deuterated DMSO), such as hydrogen bonding, the anisotropy of the solvent molecules, the polarity of the solvent, and Van der Waals interactions, are responsible for accurate quantification and integration of each proton. Additionally, ^13^C NMR was used to confirm the structure and assigned to the literature (Figure 1b) [36,37]. The carbon attached to the hydroxyl group (−***C***H_2_OH) was observed at 49.5 ppm, while the carbon attached to nitrogen (−(P=O)-N***C***H_2_−) was observed at 57.1 ppm. The carbons of the aromatic groups were observed in the range of 127.2–140.3 ppm. Heterocoupling between the C-nucleus and the P-nucleus was observed at 139.0 ppm and 140.3 ppm with a *J*_C-P_ value of 173.4 Hz. Furthermore, diethanolamine ^13^C NMR analysis was conducted in the same solvent for comparison to detect any residual traces in the product. Carbon atoms were observed at 49.5 ppm (−NCH_2_***C***H_2_OH) and 59.0 ppm (−N***C***H_2_ CH_2_OH) on the ^13^C NMR spectrum. It was found that there is no residual diethanolamine.

In Figure 1a, the inset spectrum displays the ^31^P NMR spectrum of DYM, wherein two signals are observed at 9.3 ppm and 2.2 ppm, respectively. Although the DYM structure contains one phosphorus atom, the second signal at 2.2 ppm is attributed to the potential formation of a byproduct during the reaction. It was hypothesized that the observed peak was indicative of the hydrolysis of phenyl phosphonic dichloride in the presence of residual water in diethanolamine stock, despite the stock having been dried prior to use. To substantiate this hypothesis, a model compound of phenyl phosphonic acid was synthesized under identical reaction conditions, employing triethylamine and phenyl phosphonic dichloride dissolved in D_2_O within an NMR sample tube. When the ^31^P NMR technique was applied to this model compound, a single phosphorus peak was observed at 2.2 ppm. The percentage of byproducts in the parent compound was calculated using integration values in ^31^P NMR data. The quantity of this byproduct was found to be approximately 10% by mole. It should be noted that as in the ^1^H NMR analysis mentioned above, integration values might deviate due to the improper NMR acquisition set up, which fails to collect enough scans to achieve a sufficient signal-to-noise ratio.

### 3.2. Synthesis of phosphorus possessing soy polyol (ESBO-DYM)

**Figure 2:**
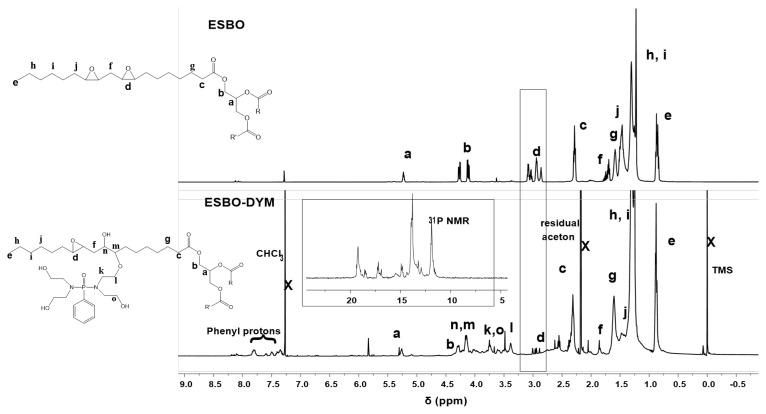
^1^H NMR spectra of a) ESBO and b) ESBO-DYM (inset spectrum is ^31^P NMR).

The ESBO-DYM polyol was obtained by catalyzing ring-opening of oxirane of phosphorus-based tetraol (DYM) and epoxy soybean oil. The structure was confirmed through ^1^H NMR, ^31^P NMR, and FTIR analyses. Figure 2 represents the ^1^H NMR spectra of the ESBO (Figure 2a) and ESBO-DYM polyol (Figure 2b). The protons attached to the oxirane ring of ESBO were observed to be in the 3.10–3.27 ppm region [6] in Figure 1a. However, following the ring-opening reaction between ESBO and DYM, the protons of the oxirane ring disappeared in Figure 2b. The introduction of aromatic groups to the ESBO structure was also confirmed by the appearance of aromatic protons in the 7.30–8.00 ppm region. The signals that appeared in the region of 3.50–3.70 ppm were attributed to the methynic protons of the functional groups (−C**H**OH) which were formed due to the ring-opening of epoxide groups [35,39,40]. Furthermore, the ester linkages of triglycerides underwent analysis to investigate whether they were affected by potential hydrolysis under the reaction conditions. However, the presence of glycerol signals and the triglyceride structure remained unaffected.

The incorporation of phosphor groups into the ESBO structure was analyzed using ^31^P {^1^H} NMR, and multiple peaks were observed between 10 ppm to 20 ppm. The presence of residual phenyl phosphonic acid residue in the DYM adduct can also participated in the ring-opening reaction of the epoxide ring, and due to the presence of this residue, different phosphorus functionalities are formed in the ESBO-DYM structure.

**Figure 3:**
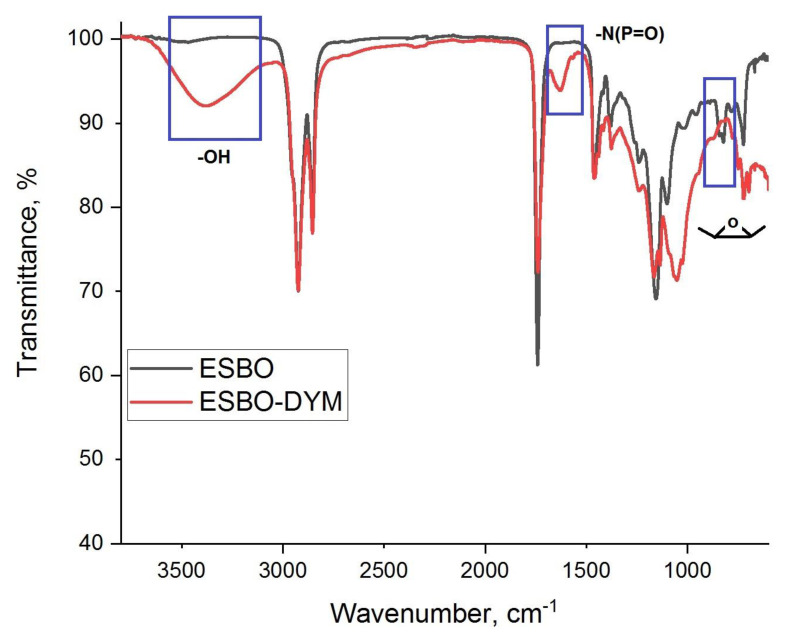
FTIR spectra of ESBO and ESBO-DYM.

For further characterization of the ESBO-DYM structure, FTIR analysis was performed. The epoxy ring stretching observed at 823 cm^−1^ [35,40] disappeared after the ring-opening reaction with tetraol (DYM) structure (Figure 3). Additionally, the vibration band attributed to the −OH stretching at 3373 cm^−1^ and the characteristic absorption band at 1661 cm^−1^ due to the phospho-amide stretching (−P(=O)N−) [26] were observed in the spectrum of the ESBO-DYM polyol. The other characteristic vibration bands were observed for ester carbonyl stretching (−C=O) at 1745 cm^−1^, 2925 cm^−1^ (−CH_2_ asymmetric stretching), and 2855 cm^−1^ (−CH_3_ symmetric stretching), respectively. The vibration band at around 1033 cm^−1^ indicates the presence of P–N–C stretching [26]. These findings prove that the targeted phosphorus and nitrogen-containing product, the ESBO-DYM polyol, was synthesized successfully.

### 3.3. The utilization of ESBO-DYM in PU foam formulation and thermal decomposition

Rigid PU foams were formed by the reaction between the synthesized biobased ESBO-DYM polyol and the polymeric MDI. PU foams were formulated using ESBO-DYM polyol at different ratios with required amounts of catalyst, water, and polyether polyol by mixing them at 21 °C which was reported in Table 1. The effect of ESBO-DYM polyol addition at different ratios to the PU foams formulation, the foam densities, and the thermal properties was investigated and compared with the properties of polyether polyol. Table 2 shows the formation times, the gelation times, the tack-free time (tapping times), and foam densities of the PU foams. It was observed that as the amount of ESBO-DYM polyol increased, the tapping times were reduced. However, the gelation time of the composition ESBO-DYM (50%) was found to be the lowest and formed a gel in 13.2 s. Foam formation time was also analyzed, and it was observed that using ESBO-DYM (100%) as a polyol exhibited behaviors that were nearly identical to those of the standard polyol (polyether polyol) and both resulted in foam formation within 7.0 s.

The density of the foams ranged from 29.1 ± 0.2 kg/m^3^ to 46.8 ± 1.2 kg/m^3^ (Table 2) and it was observed that density of the foams was decreased with the increasing ESBO-DYM content in the formulation. Notably, the foam possessing the highest amount of ESBO-DYM displayed a density of 29.1 ± 0.2 kg/m^3^. One of the main reasons of the observed decrease in density is the presence of residual moisture in the ESBO-DYM. The water in the polyol mixture reacts with an isocyanate, initially forming carbamic acid, which spontaneously decomposes to form an amine, heat, and carbon dioxide. The formation of CO_2_ gaseous product causes bubbles and creates a foam in the matrix. Thus, the density of the foam is related to the amount of water in the polyol mixture, and the presence of relatively higher amount of water molecules causes a decrease in the density of the foam [39,40]. The density of the foam also indicates the crosslinking density of matrix [41]. Presumably, the higher reactivity of commercial polyether polyol toward to isocyanate results in higher crosslinked density and forms a denser matrix.

The compressive strength of the PU foam is one of the important parameters and significantly dependent on the density of the foam. The compression strength of the standard polyether polyol-based foam was 200 kPa at 10% relative deformation. It was observed that the increasing amount of ESBO-DYM polyol content of the foams resulted in decreasing compression strength. For example, using 15% ESBO-DYM by weight in the foam formulation showed a compression value of 158 kPa under identical conditions. Hydrogen bonds between segments in urethane groups and the formation of crosslinks between molecular chains are factors affecting the mechanical properties [42,43]. Typically, the low molecular weight commercial polyether polyol (*M*_w_ = 630 g/mol), with higher primary hydroxyl value, induces more reactions between isocyanates, thus forming more urethane and urea linkages between the polymer chains. This results in a greater number of hydrogen bonds, consequently leading to higher tensile strength. Additionally, it should be noted that ESBO-DYM contains fatty alkyl chains which can serve as plasticizers, thereby increasing the flexibility of the polymer matrix.

#### 3.3.1. Thermal analysis

**Figure 4:**
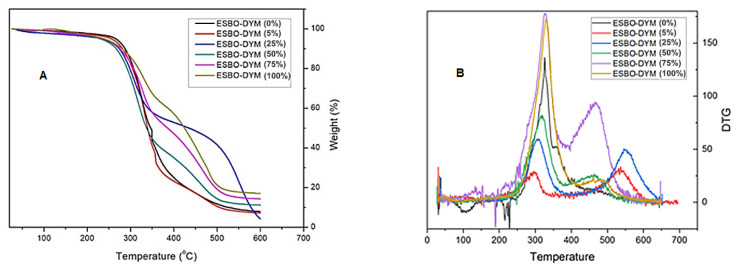
TG (a) and DTG ( b) curves of the samples (under nitrogen atmosphere).

The thermal stability of the PU foams is also an important parameter for their potential applications. Therefore, the thermal behaviors of the PU foams were investigated using TGA analysis. TGA analysis was conducted in a temperature range from 40 ºC to 600 ºC, employing a heating rate of 10 ºC/min under a nitrogen atmosphere. Figure 4 represents the corresponding TGA curves of the ESBO-DYM polyol-based PU foams. Examination of the TGA curves reveals that the decomposition of PU foams started with a minimal weight loss at around 100 °C due to the release of residual water. This was followed by two main decomposition steps. The first decomposition occurred between 280 and 350 °C due to the degradation of urethane linkages and followed by the breaking of ether bonds of polyols, which caused the formation of volatile compounds mainly carbonyl compounds, CO_2,_ and esters [44]. The second decomposition occurred above 450 °C attributed to thermo-oxidative degradation of polyurethane and aromatic groups. According to the thermograms (Figure 4a), the standard polyether polyol-based PU foam, which is absent of any P-content, had a char residue of 7.9% at the end of the cycle. The addition of 50% and 75% ESBO-DYM polyols in the foam formulation resulted in an increase in the char yield to 11.2% and 14.3%, respectively. Regarding that, the PU foam which was formulated using only ESBO-DYM (100%) polyol had the highest char residue (17%). However, surprisingly, the PU foams that were formulated using 5% and 25% ESBO-DYM had lower char residue than the standard polyether polyol-based foam which were 7.18% and 4.15%, respectively. It was predicted that the phosphor-nitrogen content of the foams would be directly related to the amount of ESBO-DYM polyol, and it would counteract the thermal instability of the triglyceride molecules, which was proven to be true at weight ratios of 50% and above. According to the thermograms, the optimum concentration of ESBO-DYM should be greater than 25% to achieve higher thermal stability. DTG curves of phosphorus-containing samples showed two peaks in the range of 250 to 600 °C due to a two-stage decomposition accordingly (Figure 4b). Generally, the first peak is attributed to the decomposition of urethane linkages and formation of isocyanates and polyols, while the second stage was assigned for further decomposition of polyols and isocyanates to generate diverse small molecules [45,46]. The degradation is single-stage in the case of ESBO-DYM (0). The phosphorus-containing samples decompose at second stage between 400 and 550 °C to generate volatile compounds and thus promote formation of the char residues. That is why the phosphorus bearing foams show a lower maximum mass loss rate compared to the standard polyether polyol-based PU foam.

The presence of phosphorus causes the formation of a char layer that insulates the polyurethane matrix from heat. It is known that phosphorus-bearing flame retardants act in condensed and vapor phases [47]. The release of the PO radicals and the development of polyphosphoric acid residue during the pyrolysis enhance the flame retardancy of the compounds [29]. The presence of nitrogen also appears to catalyze the formation of a carbon layer and synergize the flame retardant action of phosphorus on the polyurethane foam matrix. The synergistic effect of phosphorus and nitrogen-containing polyol in improving the flame retardancy of polyurethane foam was also reported [48]. Phosphorus-nitrogen-containing soy polyol can enhance the extent of crosslinked density, resulting in the formation of a stable structure under both oxidative and inert atmospheres [49,50]. TGA is not a realistic scenario for the matrix on fire; thus, LOI and UL94 analyses are conducted to investigate the flame retardancy of the foams.

#### 3.3.2. Limiting oxygen index (LOI)

The liming oxygen measurements were performed according to ASTM D2863-17. The foams were prepared in dimensions of 12 cm × 2 cm × 1 cm and placed in the test apparatus in a vertical position against the fire.

LOI of the standard polyether polyol-based PU foam was determined to be 18.3% and the LOI of 50%, 75%, and 100% ESBO-DYM polyol-based PU foams were found to be 21.8%, 22.3%, and 22.9%, respectively (Table 3). When ESBO-DYM concentration of up to 25% was used in the foam formulation, the LOI value of the foam was almost similar to that of the foam obtained with standard polyol. The flame retardancy of the foams was dramatically enhanced due to the increasing phosphorus content of the foams after reaching a certain concentration. The threshold concentration of ESO-DYM affects the LOI values as shown in the TGA analysis. This result also correlates with the reports given by Heinen et al. and Zhang et al. about phosphorylated biobased polyurethane [35,51]. Based on the results, it can be concluded that the ESBO-DYM presence in the foam exhibited a synergistic effect between phosphorus, nitrogen, and increased thermal stability.

Vertical burning tests according to the UL-94 standard (ASTM D3801-20a) were also conducted to investigate the flame retardant properties of the samples. It was observed that all the samples burnt out within a few seconds without any melt drips and resulted in UL-94 V-2 rating in the vertical test. (Table 3). The rate of the flame spreading over the foam surface was also observed.

#### 3.3.3. Morphological analysis

**Figure 5:**
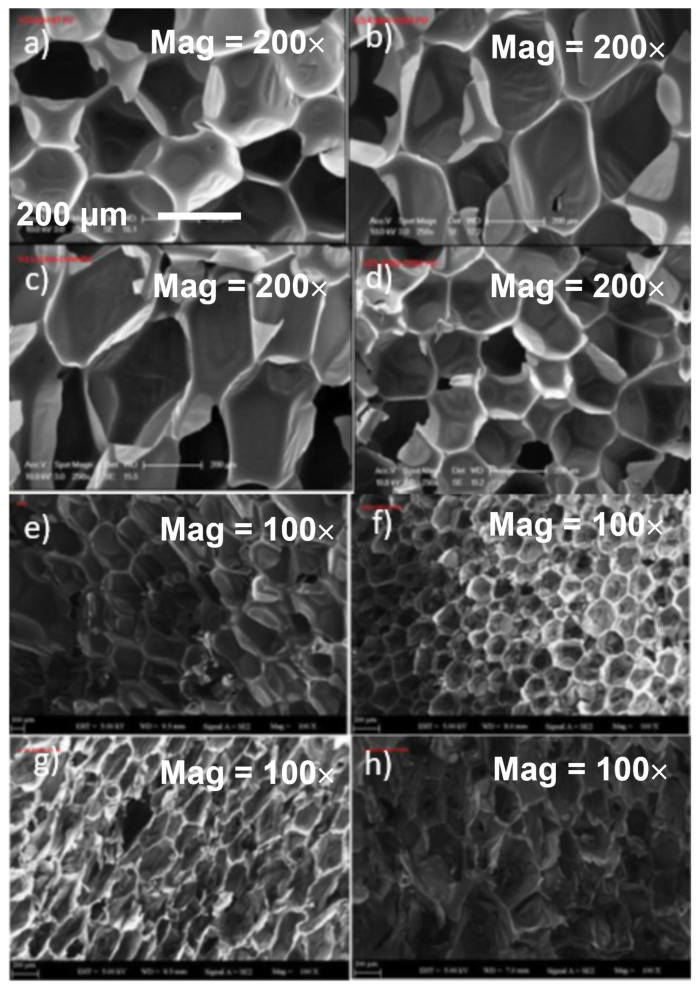
SEM images of a) and b) standard polyether polyol-based PU, c) 5% ESBO-DYM PU, d) 15% ESBO-DYM, e) 25% ESBO-DYM, f) 50% ESBO-DYM PU, g) 75% ESBO-DYM PU, h) 100% ESBO-DYM PU (magnification 100× and 200×, bar 200 μm and bar is presented in Figure 2a).

Figure 5 shows the SEM micrographs of the formulated PU foams. As depicted in Figure 5, the PU foams exhibit polygonal and elliptic cell structures. The PU foams, based on standard polyester polyol and containing 5% ESBO-DYM, have predominantly uniform cell distributions (Figures 5a and 5b). However, increasing the amount of phosphorus-nitrogen-containing soy-based polyols in the foam results in the formation of nonuniform cell structures with longer and/or smaller pores (Figures 5c and 5d). The nonuniform distribution of cells is due to microphase separation between the epoxidized soy-based polyol (ESBO-DYM) and standard polyether polyol. Silicones are commonly used as a surfactant in the formulation of polyurethane-based foam, providing nucleation, stabilization, and emulsification of the polyol mixture. The compatibility of polyol, silicone, and blowing agents results in a uniform cell structure. Conversely, the basic incompatibility of silicone types may also cause the cell structure to become nonuniform. ESBO-DYM is a type of polyol possessing long-chain alkyl groups. The incorporation of different proportions of phosphorus and nitrogen-containing soy-based polyols into the PU foam structure resulted in differences in the foam density and affected the size of the cells.

The average cell size of the standard polyether polyol-based PU foam was found to be 285.4 μm. Conversely, the PU foams formulated with 50% ESBO-DYM and 75% ESBO-DYM resulted in smaller sizes of 158.8 μm and 186.1 μm, respectively. These soy-based polyols possessed lower foam density compared with the standard polyether polyol-based foams (Table 2).

The dimensions of the samples were also investigated using a standard ruler. Foams were cut from the core as rectangular prisms with dimensions of 10 cm × 10 cm × 5 cm, then kept at −20 °C for 24 h and 70 °C for 24 h under atmospheric conditions. After 24 h, it was observed that ESBO-DYM polyol-based PU foams did not expand at these temperatures under atmospheric conditions.

#### 3.3.4. An overview of the life cycle assessment

Life-cycle assessment (LCA) is used to support decision-making for sustainable development [52]. LCA deals with the environmental impacts from raw materials to disposal that occupy the entire life cycle of a product. Nowadays, sustainability is of growing interest. LCA can be applied to analyze the environmental impact of the product, the toxic materials on soil, air, and water bodies, and the human health impact. These assessments support the decision-makers for sustainable design. In this study, soybean oil-based polyol was used in polyurethane foam formulation. The amount of phosphorus-containing soy polyol in the foam is approximately 36% by weight. The amount of soybean oil is 33% by weight in total. Soybean oil belongs to the vegetable oil class and serves as a biodegradable and environmentally friendly raw material. The presence of phosphorus in soy polyol is an essential element for the life cycle and is not a renewable resource. Global reserves are anticipated to be depleted within 75–100 years; therefore, recovery technologies should be implemented [53].

Phosphorus-bearing polyurethane matrix, at the end of its life cycle, ends up in wastewater and accumulates in sewage sludge or soils. Phosphorus (P) can be recovered after anaerobic digestion and from sewage sludge ashes, primarily in the form of magnesium ammonium phosphate or calcium phosphates. Sludge can be directly used as fertilizer on agricultural soils. However, recovering P using struvite precipitation [54] is an alternative method to recover P if it is undesirable in the use of fertilizers.

Although large amounts of energy and reactants are required to recover P from sludge or wastewater, the depletion of mineral P has become a significant concern. Additionally, the environmental impacts of nonleaching flame retardants are more significant overall. It was also presented that using biobased formulations in polyurethane foam has more significant impacts than their fossil counterparts [55].

LCA studies on PU foams have been reported [56]. However, incineration and reuse have been investigated, and recycling technology, primarily to obtain polyols, is one of the solutions for waste treatment. A life cycle assessment of polyurethane foams derived from polyols was conducted via glycolysis of polyurethane scraps [57]. Climate change, photochemical ozone formation, particulate matter, fossil fuel resource use, and foam mass required are investigated, and recycled content of 50 and 75% demonstrated superior environmental effects than other percentages.

To sum up, the use of biobased raw materials and phosphorus-based functional structure provide significant advantages for the polyurethane foam. However, conducting a detailed LCA analysis is necessary to thoroughly assess the potential environmental impact.

## 4. Conclusion

In this study, phosphorus and nitrogen-containing novel epoxidized soybean oil-based polyols (ESBO-DYM) were synthesized using commercially available compounds. ESBO-DYM was subsequently used in PU foam formulation at varying weight ratios. The synergistic effect of phosphorus and nitrogen resulted in improving the thermal properties of PU foams. TGA analysis revealed that the incorporation of 100% ESBO-DYM polyol in the foam formulation resulted in the highest char yields of 17.0%. Additionally, the LOI of the foams increased from 18.3% for standard polyether polyol to 22.9% for 100% ESBO-DYM. Introducing ESBO-DYM into the PU foam formulation resulted in lower foam density and a drop in compression strength. Furthermore, despite the foam densities decreasing with the addition of ESBO-DYM into the foam structure, the dimensional resistances of the PU foams did not alter. Biobased polyurethane foam has gained attention in various industries due to its eco-friendly nature and versatile applications. For example, biobased polyurethane foam is used in car seats, headrests, armrests, and interior panels, offering comfort and durability. It is also employed in buildings as an insulating material, improving energy efficiency by reducing heat transfer.

Considering their natural polyol composition and flame-retardant properties, it is clear that the use of phosphorus-containing soybean oil-based polyols in the production of polyurethane foam would hold high potential value for various applications.

## Figures and Tables

**Scheme:**
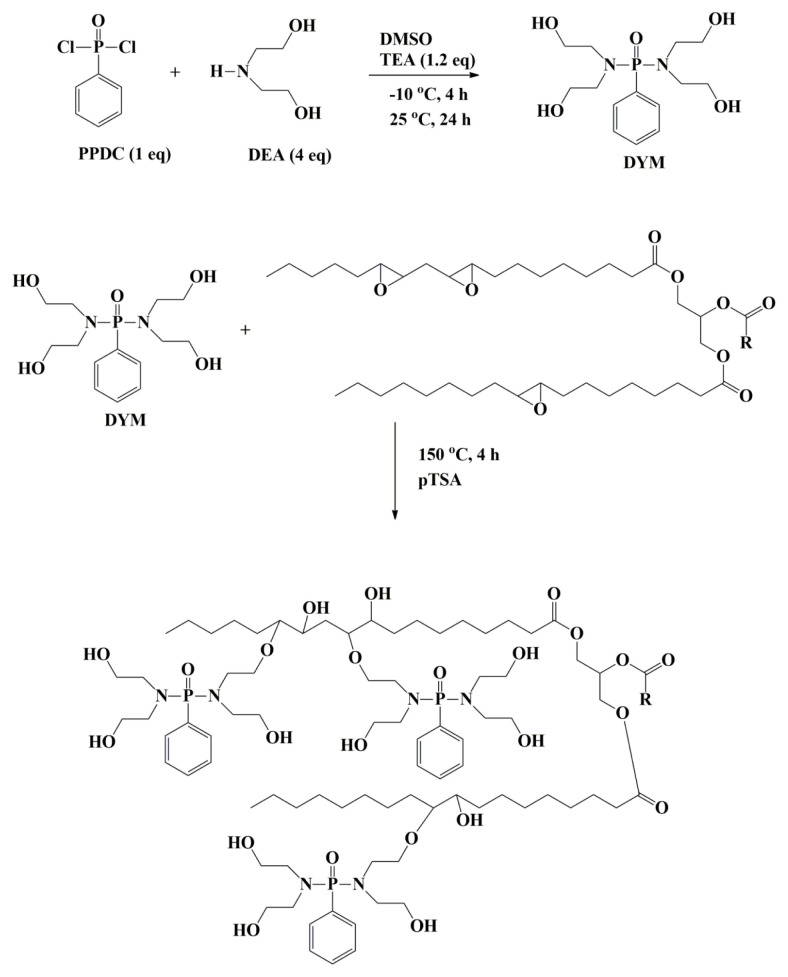
Synthetic route of phosphorus functional biobased polyol (ESBO-DYM). One substitution of the OH group of DYM to the ESBO is shown to make the schematic look better.

**Table 1:** PU foam formulation.

Polyurethane	Polyether polyol (wt.%)	ESBO-DYM (wt.%)	Silicon (wt.%)	Catalyst (wt.%) (DMCHA %6.2, BL-11 1.3%)	Water (wt.%)
Standard	70.0	0.0	1.0	7.5	2.1
ESBO-DYM (5%)	66.5	3.5	1.0	7.5	2.1
ESBO-DYM (15%)	59.5	10.5	1.0	7.5	2.1
ESBO-DYM (25%)	52.5	17.5	1.0	7.5	2.1
ESBO-DYM (50%)	35.0	35.0	1.0	7.5	2.1
ESBO-DYM (75%)	15.5	52.5	1.0	7.5	2.1
ESBO-DYM (100%)	0.0	70.0	1.0	7.5	2.1

**Table 2:** Reaction profiles and density of synthesized ESBO-DYM polyurethanes*.

Sample	Formation time (s)	Gelation time (s)	Tack-free time (s)	Density (kg/m^3^)

Standard (polyether polyol)	6.8	16.9	21.0	46.6 ± 1.2
ESBO-DYM (5%)	13.0	43.0	80.0	46.8 ± 1.2
ESBO-DYM (15%)	9.0	22.0	32.0	34.8 ± 0.8
ESBO-DYM (25%)	9.0	25.0	33.0	34.0 ± 0.6
ESBO-DYM (50%)	6.4	13.2	21.6	31.8 ± 0.8
ESBO-DYM (75%)	7.6	16.2	21.5	30.2 ± 0.4
ESBO-DYM (100%)	7.0	13.9	19.6	29.1 ± 0.2

* Duplicate analysis was performed.

**Table 3:** Limiting oxygen index (LOI) and vertical UL94 test values of the foams.

Sample^a^	LOI (%)	Vertical UL94
Standard	18.3 ± 0.2	V2
5% ESBO-DYM	18.5 ± 0.2	V2
15% ESBO-DYM	18.8 ± 0.2	V2
25% ESBO-DYM	18.8 ± 0.2	V2
50% ESBO-DYM	21.8 ± 0.2	V2
75% ESBO-DYM	22.3 ± 0.2	V2
100% ESBO-DYM	22.9 ± 0.2	V2

a Polyol content used in the polyurethane foam formulation.

b Limiting oxygen index.
